# LOW-COST SIMULATOR ASSEMBLY FOR 3-DIMENSIONAL VIDEOSURGERY TRAINING

**DOI:** 10.1590/0102-672020180001e1384

**Published:** 2018-08-16

**Authors:** Carlos Magno Queiroz da CUNHA, Douglas Marques Ferreira de LIMA, Francisco Julimar Correia de MENEZES

**Affiliations:** 1 Laboratory of Medical Skills, University of Fortaleza, Fortaleza, CE, Brazil.

**Keywords:** Education, Medical, Training, Surgery, Simulation, Educação Médica, Capacitação, Cirurgia, Simulação

## Abstract

**Background:**

Three-dimensional videosurgery is already a reality worldwide. The trainee program for this procedure should be done initially and preferably in simulators.

**Aim:**

Assemble low-cost simulator for three-dimensional videosurgery training.

**Methods:**

The simulator presented here was mounted in two parts, base and glasses. After, several stations can be inserted into the simulator for skills training in videosurgery.

**Results:**

It was possible to set up three dimensional (3D) video simulations with low cost. It has proved to be easy to assemble and allows the training surgeon of various video surgical skills.

**Conclusion:**

This equipment may be used in undergraduate programs and advanced courses for residents and surgeons. The acrylic box allows the visualization of the task executed by the tutor and even by other experienced students.

## RESUMO

**Racional:**

A videocirurgia em três dimensões já é realidade no cenário atual. O treinamento dessa habilidade deve ser feito inicialmente e preferencialmente em simuladores.

**Objetivo:**

Montar simulador de baixo custo para treinamento de videocirurgia em três dimensões.

**Métodos:**

O simulador aqui apresentado foi montado em duas partes, base e óculos. Após montagem, podem ser inseridas estações diversas no simulador para treinamento de habilidades em videocirurgia.

**Resultados:**

Foi possível montar simuladores de videocirurgia em 3D com custo baixo. Ele apresentou-se ser de fácil montagem permitindo o treinamento de várias habilidades videocirúrgicas.

**Conclusão:**

Este equipamento pode ser utilizado tanto em cursos básicos para a graduação quanto para avançados destinados a residentes e cirurgiões. A caixa de acrílico permite a visualização da tarefa executada pelo orientador/tutor e por outros alunos.

## INTRODUCTION

Since the first videosurgery in humans in 1988, this technique has grown in relation to the technology that supports it, evolving to natural orifice transluminal endoscopic surgery (NOTES) and robotic surgery. The latter and videosurgery with visualization in three dimensions provided to the surgeon the sensation of depth that previously did not exist through video^9^ ^,^ ^11^.

For the use of such technologies initially it is necessary to have the surgeon´s training. In it, for ethical reasons, it is preferable to start in simulators and preferably in models that do not use animals. It is also important to emphasize that commercial simulators usually have higher costs than those assembled by trainee for himself^3^ ^,^ ^9^ ^,^ ^10^.

Therefore observing the increasing use of this type of technology in surgery, the need for constant training, as well as the ethical embargoes involved in the use of live models, had this proposal the objective of presenting a low-cost simulator for training in three-dimensional videosurgery.

## METHOD

The study was approved by the Ethics Committee of the University of Fortaleza, Ceará, Brazil, under the number CAAE 64254316.0.0000.5052.

### Assembly of the simulator

The model described here is assembled in two parts, the base and the virtual reality glasses. The base must have aperture to accommodate the chosen eyeglasses and its cut should be of appropriate size to accommodate the device. Virtual reality glasses can be purchased or assembled. For assembly, Google® may help to explain how to do (https://vr.google.com/cardboard/manufacturers). The biconcave lenses used in the glasses can be bought in optical stores, obeying the specifications of size (diameter 34 mm) and focal length (40 mm) contained in the address described above.

**FIGURE 1:**
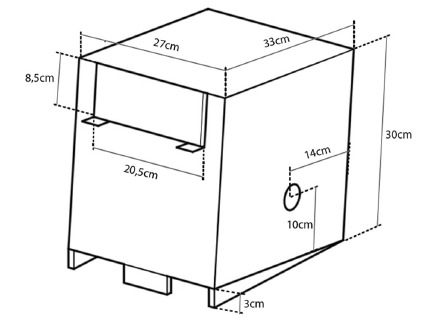
Design of the simulator: the diameter of the lateral holes must be made according to the trocars to be used

**FIGURE 2:**
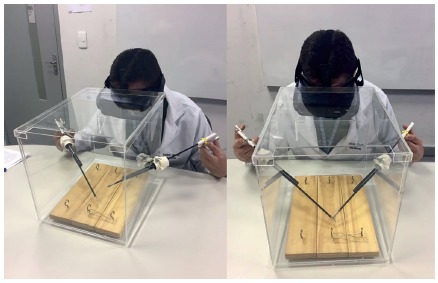
Acrylic device being used in training

The base or box must preferably be made in acrylic; in it, three openings (Figure 1) must be made for the instruments to be used, in addition to the aperture for the glasses. When using, the glasses should be attached to its appropriate opening and the trocar/forcep inserted in the holes made for them (Figure 2).

### Application

In the base is possible to be done several skills training in videolaparoscopy, such as: adaptation; visual accommodation and manipulation of tweezers in 3D environment by displacement of elastic alloys on a wooden platform (Figure 2); adjustment of the needle in the needle holder; realization of knots and sutures in 3D environment with models performed by trainees themselves or commercially sold.

## RESULTS

With this project, it was possible to set up simulator of videosurgery in three dimensions with cost that can be very low, depending on the need of the user and the available resources. This model, in addition to being easy to assemble and allowing the training of various video-surgical skills, may be used in basic undergraduate and advanced courses for residents and surgeons. These authors suggest the use of acrylic box instead other material, as it allows the outside visualization by the guiding/tutor of the task being performed and/or by other experienced/novice students.

## DISCUSSION

This model, besides being easy to assemble and allowing training in several video-surgical skills, can be used in both basic and advanced courses. Although the base can be made of several materials, the acrylic was chosen because it allows the visualization of the task being executed by the guiding/tutor and also seen by other students.

All training involves a learning curve. This curve in the videosurgery is of great importance since when it is better trained the operative time and tissue trauma decrease considerably, generating benefits for the patient. Therefore training using means that do not involve patients is justified. With this prerogative in mind, it is proposed that the training be done in simulators using artificial models or in isolated organs/parts that simulate the surgical needs and thus be more realistic. In this way, the surgeon’s ability to transform the future operative procedure with greater patient safety is promoted^1^ ^,^ ^6^ ^,^ ^2^. There is a tendency in more current training to use synthetic devices - generally reusable - easy to access and storage, to not enter into the bioethical merit when using biological material for training^3^ ^,^ ^4^ ^,^ ^5^.

In the scope of graduation, the teaching of videosurgery is already a reality and has been growing along with the use of technology with simulators, since the student has to be prepared for the changes of the current and future scenario^7^ ^,^ ^8^.

Therefore models such as this one addressed to the technological vanguard, which are operations in 3D, will be increasingly useful in teaching undergraduate medical and continuing surgical education.

## CONCLUSION

This simulator model for 3D operation, made with simple materials and low cost, is suitable for several training modalities in video surgery.
